# The PASE Study: The Prevalence and Associations of Bendopnoea in Patients with Pneumothorax

**DOI:** 10.3390/arm94050068

**Published:** 2026-09-22

**Authors:** Bianca M. Iacopetta, Niranjan Ananda Setty, Chloe Charlesworth, Michaela Donaghy, Carolyn J. Peddle-McIntyre, Ai Ling Tan, Y. C. Gary Lee

**Affiliations:** 1 Exercise Medicine Research Institute, Edith Cowan University, Joondalup, Perth, WA 6027, Australia; biacopet@our.ecu.edu.au (B.M.I.);; 2 Pleural Medicine Unit, Institute for Respiratory Health, Nedlands, Perth, WA 6009, Australia; 3 Centre for Innovative Pleural Research, Sir Charles Gairdner Hospital, Nedlands, Perth, WA 6009, Australia; 4 Department of Respiratory Medicine, Sir Charles Gairdner Hospital, Nedlands, Perth, WA 6009, Australia; 5 School of Medicine, University of Western Australia, Crawley, Perth, WA 6009, Australia

**Keywords:** pneumothorax, breathlessness, bendopnoea

## Abstract

**Highlights:**

**What are the main findings?**

- This is the first study to identify bendopnoea as a symptom in patients with pneumothorax.
- In patients with pneumothorax, bendopnoea is associated with worse overall breathlessness and showed a signal towards worse chest pain.

**What are the implications of the main findings?**

- Clinicians should recognise that bendopnoea is a common symptom experienced by patients with pneumothorax.
- If patients with pneumothorax experience bendopnoea, clinicians can explain that it is a symptom associated with the pneumothorax which should resolve with resolution of the pneumothorax.

**Abstract:**

**Background:** We recently established that bendopnoea (breathlessness upon bending forward) is common with pleural effusion; however, it has never been reported or investigated in patients with pneumothorax. This exploratory study investigated the prevalence and clinical relevance of bendopnoea in pneumothorax. **Methods:** Patients with pneumothorax of any aetiology or size were eligible. The presence of bendopnoea and time-to-bendopnoea was measured by bending the patient forward for 60 s and was assessed at baseline and post lung re-expansion. **Results:** Fifty patients (median age 48 years; 78% male) with primary spontaneous (48%), secondary spontaneous (24%) or iatrogenic (28%) pneumothorax were included. Pneumothorax size ranged from 7 to 99% of the hemithorax on chest radiographs. Most patients (74%) received conservative management. On a questionnaire, 48% (24/50) patients reported they experienced bendopnoea which “impacted daily life”. When bent forward, 32% (16/50) developed bendopnoea within 60 s. Patients with bendopnoea reported more breathlessness than those without bendopnoea on a 100 mm Visual Analogue Scale (VAS): median 46 [Q1–Q3 = 25–54] vs. 12 [3–32] mm, respectively, *p* = 0.0089; median difference −23 mm (95% CI −35 to −6). Those with bendopnoea showed a signal with worse chest pain on a 100 mm VAS for pain: median 28 [13–48] vs. 10 [2–42] mm, *p* = 0.0528; median difference −15 mm (95% CI −27 to 0). Shorter time-to-bendopnoea (i.e., worse bendopnoea) correlated with worse breathlessness (r_s_ = −0.38, 95% CI −0.6 to −0.1; *p* = 0.0072) and more severe chest pain (r_s_ = −0.29, 95% CI −0.5 to −0.007; *p* = 0.039). **Conclusions:** Bendopnoea can occur with pneumothorax, correlates with breathlessness and demonstrates a signal with worse chest pain.

## 1. Introduction

Pneumothorax is a common condition, accounting for approximately 14.1 hospitalisations per 100,000 people each year in England [1]. Breathlessness and chest pain are the most common symptoms; however, symptom severity varies amongst individuals. Bendopnoea (breathlessness upon bending forward) is a recently recognised symptom for several cardiac [2,3,4,5] and respiratory [4,6,7,8] diseases and is associated with worse morbidity and mortality. The initial characterisation of bendopnoea defined the symptom as shortness of breath occurring within 30 s of bending forward while seated [2]. Subsequent studies have extended the assessment period to 60 s [5,8], recognising that breathlessness may take longer to develop during sustained forward bending. The first study to characterise bendopnoea reported the symptom was prevalent in 28% of patients with advanced heart failure [2]. Other studies in heart failure populations have reported prevalence estimates ranging from 18% to 49% [3,4,5]. Evidence of bendopnoea in respiratory disease is emerging, with recent studies reporting prevalence of 52% in chronic obstructive pulmonary disease (COPD) [7], 47% in pulmonary arterial hypertension [4], and 35% in obstructive sleep apnoea [6]. Our recent study of pleural effusion y found that bendopnoea was prevalent in 56% of patients and was associated with larger effusions, worse breathlessness and poorer exercise tolerance [8]. Bendopnoea can help predict patients’ functional improvement upon pleural fluid drainage [8]. Bendopnoea has never been investigated in patients with pneumothorax.

The Pneumothorax And Symptom Evaluation (PASE) study was an exploratory study and the first to evaluate the prevalence of bendopnoea in patients with pneumothorax and to explore its clinical relevance including its relationship with symptoms (pain and breathlessness) and size of pneumothorax.

## 2. Materials and Methods

The protocol of the PASE study has been published [9] and is summarised below.

This was a prospective, cross-sectional study which recruited 50 participants with a new diagnosis of unilateral spontaneous (primary or secondary) pneumothorax or iatrogenic pneumothorax referred to the Pleural Service at Sir Charles Gairdner Hospital (SCGH), Perth, Western Australia. Patients were recruited between April 2024 and September 2025. Patients included were consecutive in- and out-patients with a new diagnosis of unilateral pneumothorax who were referred for assessment [9]. Patients were excluded if they had a simultaneous pleural effusion larger than 1 cm, were haemodynamically unstable (characterised by blood pressure less than 90 mmHg systolic, a heart rate that equals or exceeds systolic blood pressure mmHg in beats per minute, a respiration rate exceeding 30 breaths per minute or a SpO2 less than 90% on room air), were under 18 years of age, had mechanical limitations that prevented forward bending (e.g., spinal conditions), were pregnant or lactating, or were unable to provide informed consent or comply with the study protocol [9].

This sample size was considered feasible within the proposed timeframe and sufficient to provide a reasonable representation of different pneumothorax types and sizes of lung collapse. Written informed consent was provided by all participants. Ethics approval for the study was granted by the Sir Charles Gairdner and Osborne Park Health Care Group Human Research Ethics Committee (RGS0000006354) and is registered (ACTRN12623001109695).

The primary outcome measured bendopnoea employing a 60-second bend test (60s-BT) [2,5,8,10]. Patients were seated upright in a chair with feet touching the floor. They were instructed to bend forward and aim to touch their ankles. The position was maintained for a maximum of 60 s and patients were told to inform the investigator as soon as breathlessness occurred. The presence of bendopnoea (yes/no) and time-to-bendopnoea (up to 60 s) were recorded [8]. There is currently no established diagnostic significance or validated threshold for time-to-bendopnoea. Secondary outcomes included a bendopnoea questionnaire [5] which assessed the impact of breathlessness when bending forward on day-to-day activities since symptom onset. Responses were categorised as not at all, mildly, moderately, or severely affected. The 60s-BT and (adapted) questionnaire were both previously employed to measure bendopnoea presence and severity in patients with pleural effusions [8]. Breathlessness was measured using a validated 100 mm Visual Analogue Scale, VAS [11], commonly employed in pleural studies [11,12]. Chest pain was likewise captured using a 100 mm VAS [13]. Baseline demographical information and the size of the pneumothorax on chest radiographs according to the Light index [14] were also recorded. The Light index was calculated using the standard radiographic measurements of the collapsed lung and hemithorax to estimate the percentage of lung collapse [14]. Assessments were conducted at baseline and, where applicable, upon full lung re-expansion.

Statistical analyses were conducted using GraphPad Prism v.11 (GraphPad, Boston, MA, USA). Normality was assessed using the Shapiro–Wilk test. Data are summarised as counts and percentages and medians with interquartile boundaries [Q1–Q3]. Mann–Whitney tests were used to compare median outcome measure scores between patients with and without bendopnoea on 60s-BT at baseline. Spearman’s rank correlation coefficients (r_s_) were calculated to examine the associations between baseline bendopnoea severity (defined as time-to-bendopnoea during forward bending) and breathlessness and chest pain using VAS scores. The 95% confidence intervals (CIs) were reported for median differences and correlation coefficients.

## 3. Results

Fifty patients were included in the study (median age 48 years [22–68]; 78% male), as shown in Table 1 and Figure 1. Pneumothoraces were predominantly right sided (60%), with the size of collapse ranging from 7 to 99% of the hemithorax on chest radiographs, as shown in Table 2. The study population comprised all common types of pneumothorax: primary spontaneous pneumothorax (PSP, 48%), secondary spontaneous pneumothorax (SSP, 24%) and iatrogenic pneumothorax (28%). The most common causes of SSP were COPD (33%) and cystic lung disease (25%).

### 3.1. Self-Reported Impact of Bendopnoea on Daily Living

Responses to the bendopnoea questionnaire indicated that 24/50 (48%) of patients experienced bendopnoea which ‘impacted their daily lives’ since the onset of pneumothorax; 18/50 (36%) reported ‘no’ bendopnoea and 8/50 (16%) ‘don’t know’. Of those who reported experiencing bendopnoea, 13/24 (54%) indicated a ‘moderate’ or ‘severe’ impact on their daily activities.

### 3.2. Bendopnoea Prevalence on 60s-BT

On the 60s-BT, 16/50 (32%) of patients developed bendopnoea within 60 s of bending forward. Patients with iatrogenic pneumothorax had the highest proportion of patients who developed bendopnoea 6/14 (43%), followed by PSP 7/24 (29%) and 3/12 (25%) of those with SSP. Within the subset that developed bendopnoea, the median time-to-bendopnoea was 11 [5–17] seconds in SSP, compared with 24 [10–55] seconds in PSP and 33 [15–51] seconds in those with iatrogenic pneumothorax.

### 3.3. Bendopnoea’s Clinical Relevance and Associations

Higher levels of breathlessness were observed in patients with bendopnoea compared to those without bendopnoea: median VAS score 46 [25–54] vs. 12 [3–32] mm respectively, *p* = 0.0089; median difference −23 mm (95% CI −35 to −6), as shown in Figure 2. Shorter time-to-bendopnoea (i.e., worse bendopnoea) correlated with worse breathlessness (r_s_ = −0.38, 95% CI −0.6 to −0.1; *p* = 0.0072).

Patients with bendopnoea had worse chest pain than those without bendopnoea: median VAS score 28 [13–48] vs. 10 [2–42] mm respectively, although it did not reach statistical significance, *p* = 0.0528; median difference −15 mm (95% CI −27 to 0), as shown in Figure 3. Shorter time-to-bendopnoea (i.e., worse bendopnoea) correlated with more severe chest pain (r_s_ = −0.29, 95% CI −0.5 to −0.007; *p* = 0.039).

The subgroups of pneumothorax types and size were small and did not show a relationship with the presence or severity of bendopnoea, as shown in Figure 4. Additionally, there was no correlation between bendopnoea and Body Mass Index (BMI).

### 3.4. Bendopnoea Post Lung Re-Expansion

There were 45/50 (90%) patients who achieved full lung re-expansion after a median of 25 days (17–30), and 29/50 (58%) completed post lung-expansion assessment. Reasons why patients did not complete post lung-expansion are documented in Figure 1. There were no statistically significant differences in baseline characteristics, scores for breathlessness or chest pain between patients who completed post lung-expansion assessment and those lost to follow-up. Of the 29 patients with post lung-expansion, 13/29 (45%) reported bendopnoea on the questionnaire at baseline and after lung re-expansion 10/29 (34%) reported persistent bendopnoea that impacted their daily lives. Using the 60s-BT bendopnoea was present in 9/29 (31%) at baseline and 5/29 (17%) had persistent bendopnoea after lung re-expansion. This included three patients with PSP, one each with SSP and iatrogenic pneumothorax. There was no statistically significant improvement in time-to-bendopnoea between pre and post lung-expansion.

## 4. Discussion

The PASE study is the first to explore and report bendopnoea as a novel symptom in pneumothorax. Our results indicate bendopnoea is a symptom commonly experienced by this cohort and impacts daily lives. Bendopnoea was present in a third of patients and was associated with worse overall breathlessness and showed a signal towards increased chest pain. Clinicians should recognise bendopnoea as a symptom in pneumothorax.

In the current study, bendopnoea was present in one third of patients during the 60 s bend test; however, half of patients reported that bendopnoea affected their daily lives. It is possible that a longer duration of bending may provoke bendopnoea in more patients, particularly if the test incorporated activities that replicate real-life movements involving bending forward.

Bendopnoea is an increasingly appreciated symptom in pleural diseases. Our recently published Pleural Effusion And Symptom Evaluation (PLEASE)-3 study was the first to establish bendopnoea as a common symptom among patients with pleural effusion [8]. It also demonstrated that bendopnoea was associated with worse breathlessness and decreased exercise tolerance at baseline. Patients with bendopnoea derived more significant benefits in exercise tolerance from drainage of pleural fluid [8].

In both pleural effusion and pneumothorax, the pleural cavity must expand to accommodate the fluid and air respectively. The two conditions differ, however, in that pleural fluid is heavy (often in litres and thus kilograms), unlike air. Hence it is interesting to assess if bendopnoea occurs in pneumothorax. Indeed, in the current study, we found that bendopnoea was common in the pneumothorax population. Its presence and severity of bendopnoea did correlate with a higher level of breathlessness and demonstrated a signal towards worse chest pain, similar to our findings in pleural effusion.

With pleural effusion, it is postulated that the fluid impairs hemidiaphragm mechanics [15] and results in breathlessness [8]. In PLEASE-3, bendopnoea was related to the size of the effusion and was largely reversible once the fluid was drained [8]. It is therefore intriguing that in this current study, the size of the pneumothorax did not correlate with bendopnoea. This suggests that the diaphragmatic effect from the weight of fluid is not the only mechanism at play. Whether other aspects of diaphragmatic mechanics or pleural pressure changes contribute to the experience of bendopnoea in patients with pneumothorax remains unknown.

The underlying pathophysiological process of bendopnoea is likely to be multi-factorial [2,8,10]. In our study, physiological mechanisms associated with patients’ underlying cardiac and respiratory diseases may have contributed to the prevalence and associations of bendopnoea. Increased left ventricular filling pressures which further increases when bending may explain bendopnoea in cardiac failure [2]. This may have contributed to some of the patients in our study with cardiac comorbidities. People with chronic obstructive pulmonary disease and/or pulmonary hypertension have reported bendopnoea and may explain why patients in our study with SSP experienced the most severe bendopnoea (i.e., the shortest time-to-bendopnoea). Dynamic hyperinflation, particularly in those with SSP, may also contribute to patients’ experience of bendopnoea. Mechanical disadvantages associated with bending forward include increase in venous return and concurrent reduction in intrathoracic space and lung expansion due to compression of the hemidiaphragm [16,17]. These effects add to those from reduced lung volumes in pneumothorax.

Altered heart-lung mechanics during bending has been suggested as a contributor to bendopnoea in pulmonary arterial hypertension [10]. Increased venous return competing with restricted lung expansion is likely worsened by collapsed lung [2]. Additionally, impaired respiratory muscle function and respiratory muscle loading due to gravity when bending may be contribute to bendopnoea [8]. This hypothesis is plausible in pneumothorax, as patients may already have an increased work of breathing due to pain and breathlessness.

This study has limitations. First, this is a single-centre study which limits generalisability of the results. Second, this pilot cohort is underpowered to detect differences between pneumothorax subgroups. The study was designed to recruit consecutive patients and had minimal exclusion criteria to minimise selection bias. Third, the 60s-BT to assess bendopnoea required self-reporting of symptoms. Therefore, results could have been influenced by the Hawthorne effect [18]. This refers to changes in patients’ behaviour resulting from their awareness of being observed or participating in a study. Nonetheless, correlation of the time-to-bendopnoea with objective parameters (e.g., breathlessness and chest pain) suggest that it is a reliable observation. Fourth, the potential impact of the patients who were lost to follow up and did not complete the post lung-expansion assessments may have introduced bias. Fifth, subgroups were too small to complete multivariant analyses to determine the predictors of bendopnoea presence and factors for bendopnoea resolution. Additionally, the subgroups of pneumothorax management types were too small to compare bendopnoea post lung-expansion. Future studies that are adequately powered to assess these outcomes are required. Large cohort studies are required to investigate the clinical relevance of bendopnoea, including whether it can predict better symptomatic response to drainage procedures or its potential value in outpatient monitoring.

Bendopnoea is a novel symptom to be recognised in patients with pneumothorax. In the present study, bendopnoea was present in a third of patients and its presence correlated with worse breathlessness and showed a signal towards worse chest pain. It was not associated with the size of the pneumothorax. The pathophysiological basis of bendopnoea in patients with pleural diseases should be further explored.

## Figures and Tables

**Figure 1:**
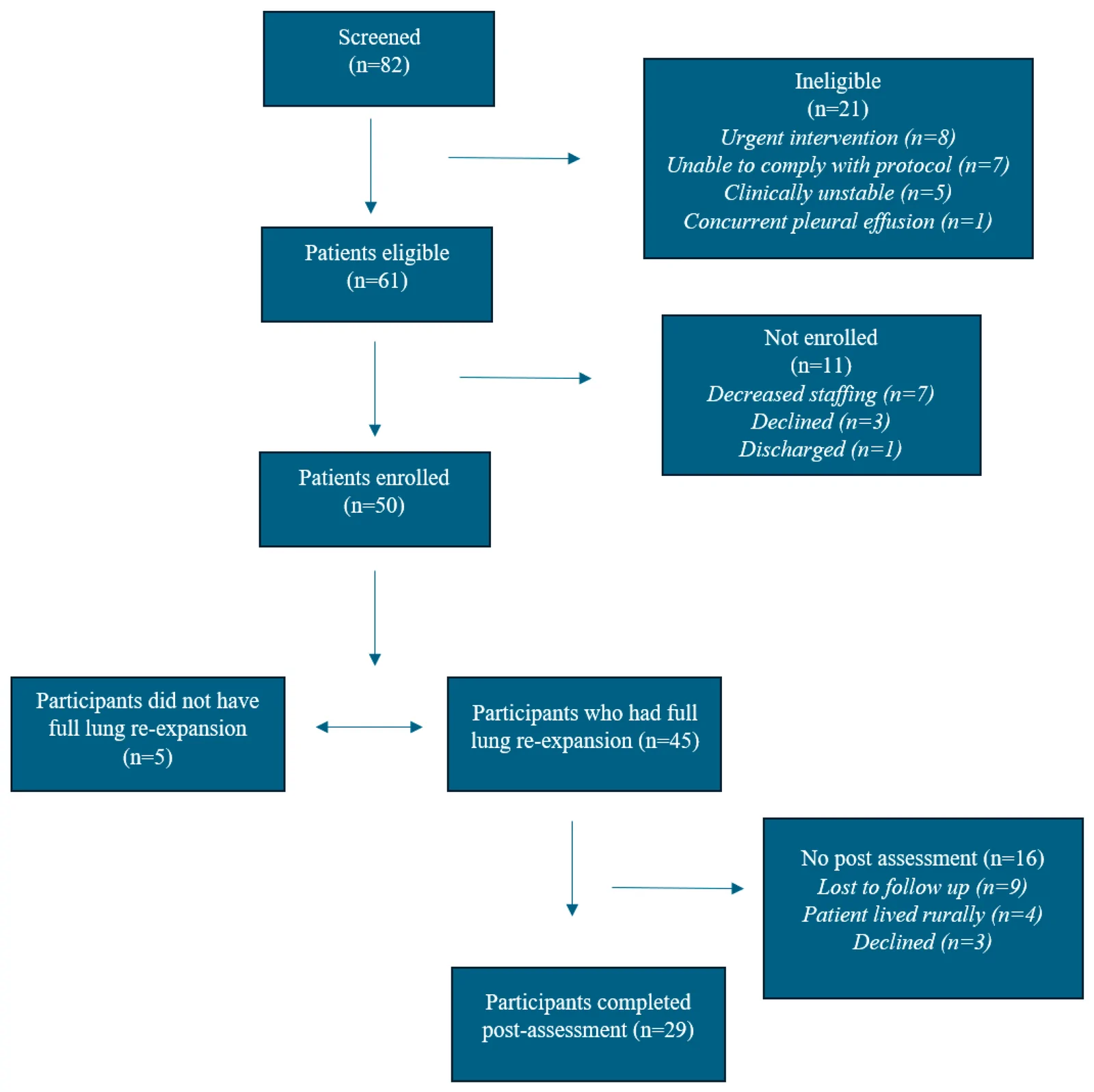
Participation flow chart.

**Figure 2:**
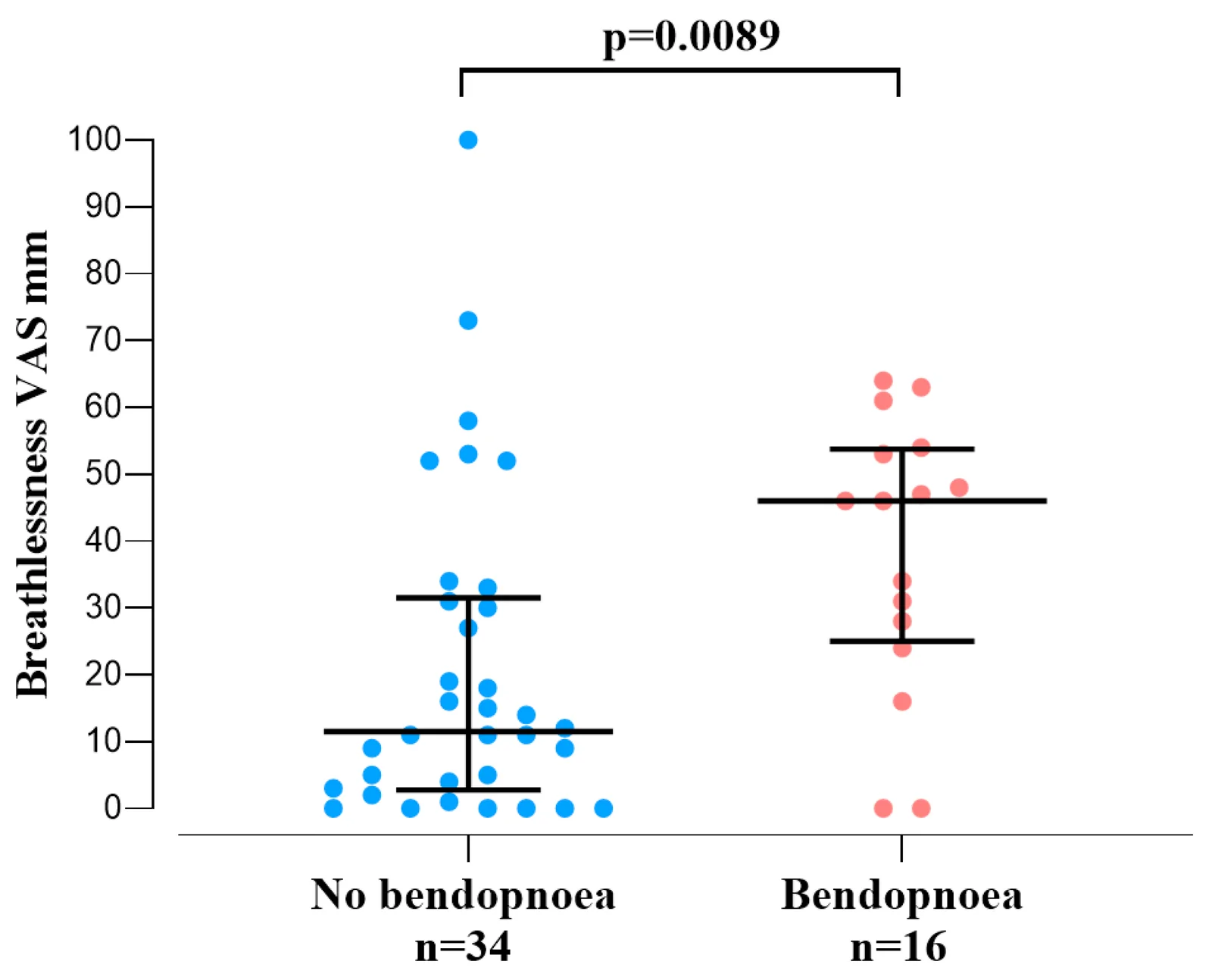
Differences in breathlessness (VAS) score for those with and without bendopnoea on the 60s-BT (n = 50).

**Figure 3:**
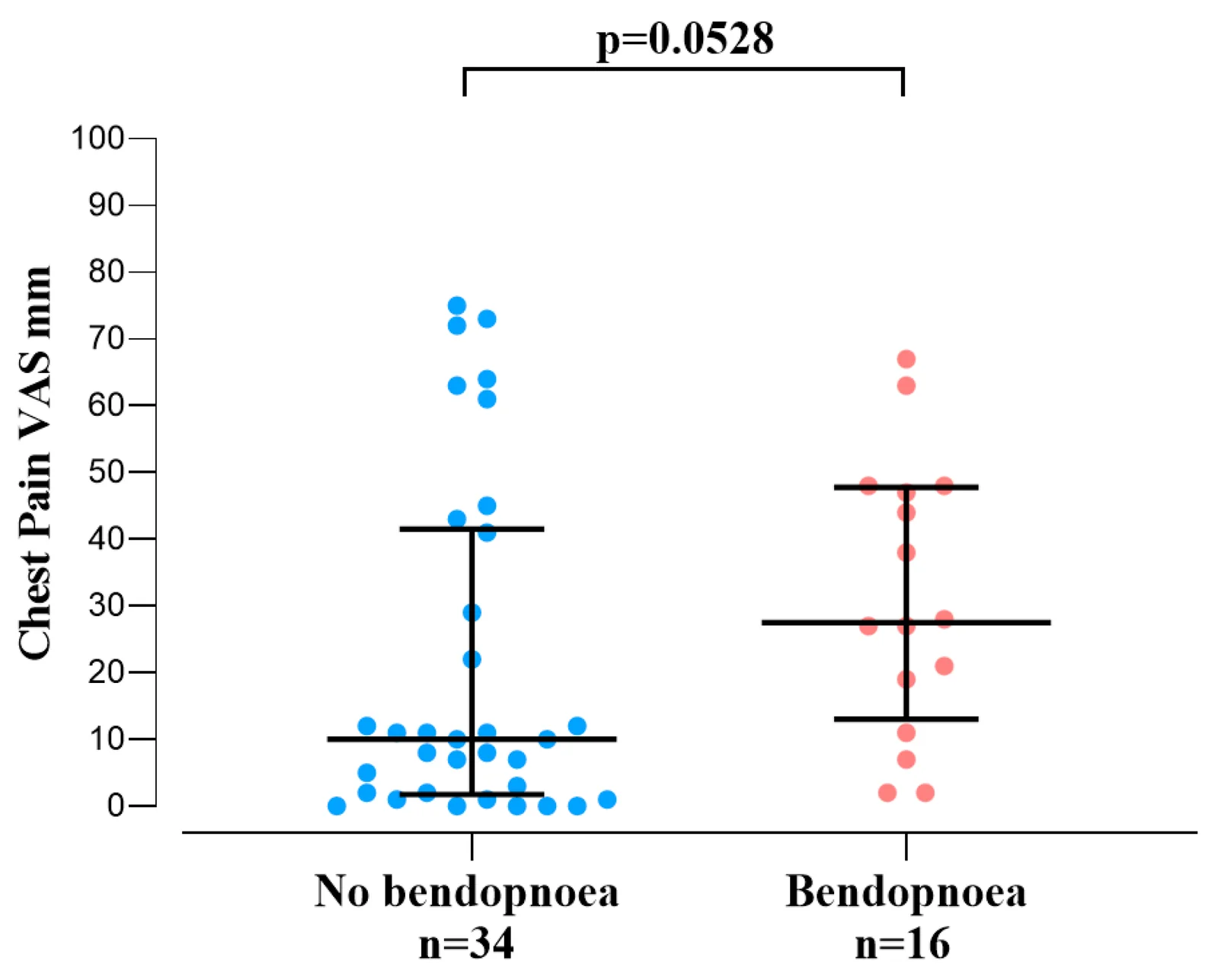
Differences in chest pain (VAS) score for those with and without bendopnoea on the 60s-BT (n = 50).

**Figure 4:**
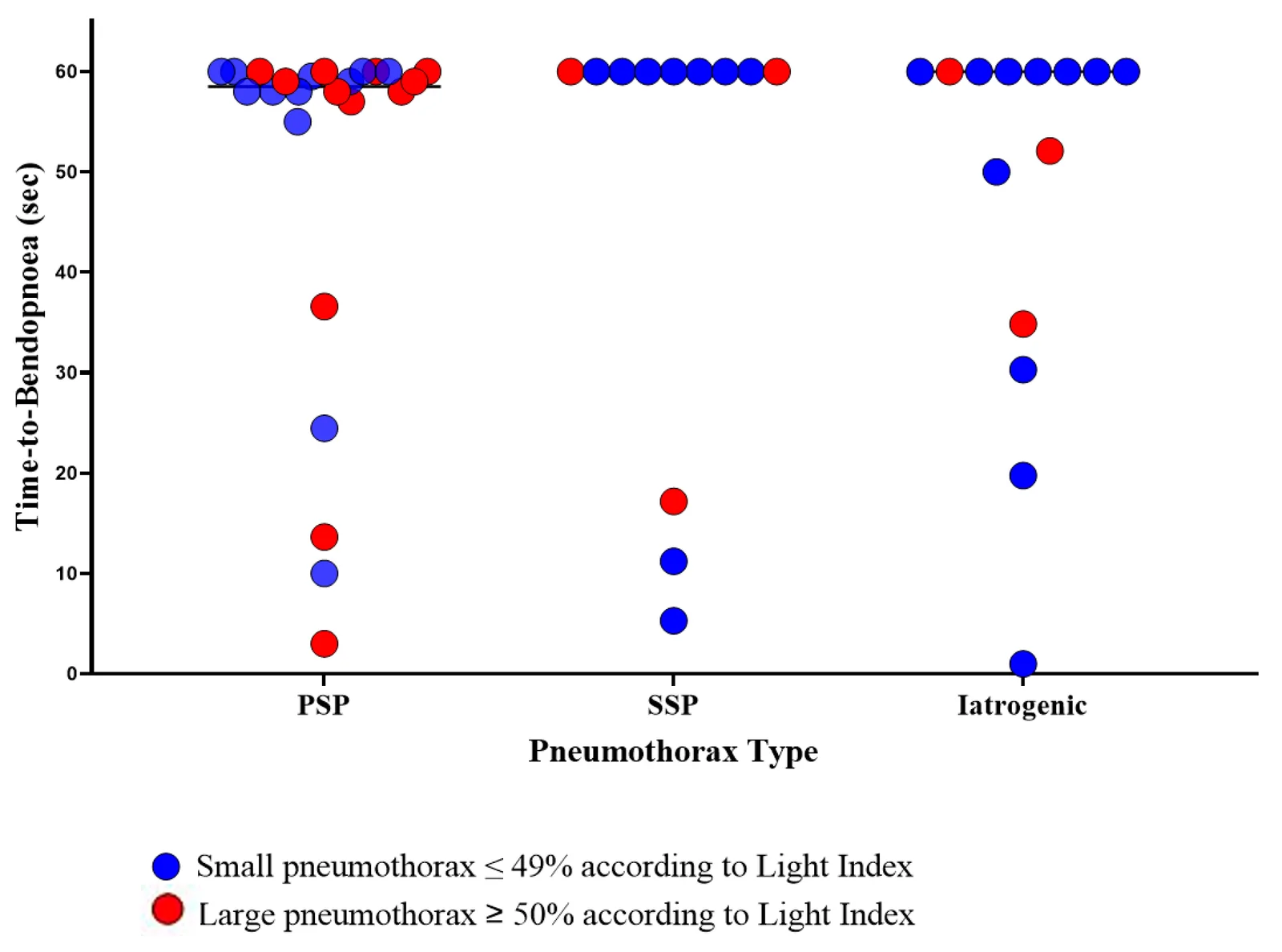
Relationship between time-to-bendopnoea and pneumothorax size/type (n = 50).

**Table 1:** Baseline characteristics (n = 50 unless otherwise stated).

Clinical Characteristics	N (%)
Male	39 (78%)
Age *	48 [22–68]
Body Mass Index (BMI) * (n = 42)	22 [20–26]
Smoking Status	
Never	21 (42%)
Current	12 (24%)
Former	17 (34%)
Pack Years *	20 [10–30]
ECOG	
0	32 (64%)
1	12 (24%)
2	3 (6%)
3	3 (6%)
4	0
Comorbidities	
Cardiac	20 (40%)
Respiratory	16 (32%)
Malignancy	11 (22%)
Mental Health	6 (12%)
Other	7 (14%)
Previous Pneumothorax	10 (20%)
Previous Pleural Procedure	5 (10%)
Breathlessness VAS Score *^	19 [5–47]
Chest Pain VAS Score *^	12 [3–44]

* Median [Q1–Q3]; ^ lower scores indicate less severe symptoms and higher scores indicate greater symptom severity. ECOG, Eastern Cooperative Oncology Group performance status.

**Table 2:** Baseline pneumothorax characteristics (n = 50 unless otherwise stated).

Pneumothorax Characteristics	N (%)
Side of Pneumothorax	
Right	30 (60%)
Left	20 (40%)
Size of Pneumothorax (n = 48) (%)	
Light index *	49 [37–75]
Type of Pneumothorax	
Primary Spontaneous	24 (48%)
Iatrogenic	14 (28%)
Secondary Spontaneous	12 (24%)
Management	
Conservative	37 (74%)
Surgery	8 (16%)
Chest Tube Placement	5 (10%)
Achieved total lung re-expansion	38 (76%)

* Median [Q1–Q3].

## Data Availability

De-identified individual participant data will be available to the scientific community with as few restrictions as possible while retaining exclusive use until publication of major outcomes. The data presented in this study are available on request from the corresponding author.

## Abbreviations

The following abbreviations are used in this manuscript:

60s-BT	60-second Bend Test
VAS	Visual Analogue Scale
PSP	Primary Spontaneous Pneumothorax
SSP	Secondary Spontaneous Pneumothorax
COPD	Chronic Obstructive Pulmonary Disease
